# Typology of patients with behavioral addictions or eating disorders during a one-year period of care: Exploring similarities of trajectory using growth mixture modeling coupled with latent class analysis

**DOI:** 10.1371/journal.pone.0207398

**Published:** 2018-11-14

**Authors:** Marion Montourcy, Jean-Benoit Hardouin, Julie Caillon, Juliette Leboucher, Morgane Rousselet, Marie Grall-Bronnec, Gaëlle Challet-Bouju

**Affiliations:** 1 Université de Nantes, Université de Tours, UMR INSERM 1246 SHERE, Nantes, France; 2 CHU Nantes, Addictology and Psychiatry Department, Nantes, France; Yale University, UNITED STATES

## Abstract

**Objectives:**

Addictions are not restricted to substance-use disorders, and it is now widely recognized that they also include behavioral addictions. Certain individuals with eating disorders also experiment their disorder as an addiction. The objective was to identify typologies of patients presenting with various behavioral addictions or eating disorders according to their evolution within the framework of care, and to specify the factors associated with the differential clinical trajectories.

**Methods:**

We included 302 patients presenting with problem gambling, sexual addiction, compulsive buying, excessive videogame use or eating disorders. The patients completed a multiaxial assessment through a face-to-face structured interview and self-administered questionnaires, including sociodemographic and addiction-related characteristics, psychiatric and addictive comorbidities and several psychological characteristics. The assessment was performed at inclusion and then repeated after 6 and 12 months. The statistical analysis included a combination of growth mixture models and latent class analysis.

**Results:**

We identified five classes of patients with different profiles related to their trajectories during a one-year period of specialized care: “complex patients”, “patients with impulsive psychological functioning”, “patients with cooperative psychological functioning”, “patients with immature psychological functioning,” and “patients with resilient psychological functioning”.

**Conclusions:**

The typology obtained brings interesting findings to propose patient-centered care strategies adapted to these disorders. Because the typology was independent from the type of disorder, it supports the general concept of behavioral addictions, and the similarities between eating disorders and behavioral addictions. The relevance of this model should be further examined in future studies.

## Introduction

Addictions are not restricted to substance use disorders, and it is now widely recognized that they also include behavioral addictions (BAs), such as gambling disorder, sexual addiction, or compulsive buying [1, 2]. The inclusion of excessive videogame use under BA has been debated [3], even if the proposed diagnostic criteria for Internet Gaming Disorder in the DSM-5 largely overlap with the concept of addiction [4]. Some authors have also noted similarities between BAs and eating disorders (ED), [5–7]. Indeed, certain individuals with an ED experience their disorder as an addiction and share the same addictive characteristics as individuals with substance-related disorders [5].

A BA, but also more largely all addictions, can be defined as a “*process whereby a behavior*, *that can function both to produce pleasure and to provide escape from internal discomfort*, *is employed in a pattern characterized by recurrent failure to control the behavior and continuation of the behavior despite significant negative consequences*” [8]. Every addiction is characterized by the loss of control over the behavior, leading to the abandonment of all other personal or business investments for the exclusive benefit of the addictive behavior. The addiction then becomes the center of the addict's existence, exposing him secondarily to multiple and severe types of damage (suicide, debt, isolation, etc.). Such definition may also overlap with EDs [5].

Addictions and EDs are characterized by a long-term evolution, which can be very different according to the various profiles of patients [1]. Treatment failures and relapses are frequent [1, 9]. In this context, it seems interesting to study the differential trajectories of patients treated for a BA or an ED. Such studies with the objective of explaining changes in the patients’ status (with or without problems) or the stability of the disorder over time exist [10–13]. However, studies investigating typologies of patients according to their evolution within the framework of care are very rare or even nonexistent. Moreover, to date, trajectory studies grouping together different forms of BAs and EDs do not exist.

The objective of the present study was to characterize the typologies of patients presenting with various BAs or EDs according to their evolution within the framework of care, without *a priori* distinguishing them according to their disorder. The aim was to identify typical trajectories and to specify the factors associated with the differential clinical evolutions. Identifying such care trajectories among a large sample of patients presenting with BAs or EDs (whatever the disorder) would allow us to highlight either: (i) a common care trajectory for all studied disorders (both BAs and EDs): such result would support the similarities between BAs and EDs, making it possible to envisage common therapeutic tracks useful for all patients; or (ii) disorder-dependent profiles, with a distinct trajectory for each type of disorder, justifying individualized care by disorder; or (iii) different trajectories not linked to the type of disorder but, rather, based on patient profiles (profiles of personality, psychiatric comorbidities, etc.): such result would also support the similarities between the disorders and the use of common therapeutic tracks independently of the disorder, but also implies to use specific care targeted for each profile (personalized medicine).

## Material and methods

### Participants

The participants were recruited as part of the EVALADD (EVALuation of behavioral ADDictions) cohort (NCT01248767), which consists of a prospective follow-up of patients seeking treatment for either a BA or an ED at the Nantes University Hospital (France). Our addictology department is a reference center for both BAs and EDs in France. The inclusion of EDs in the EVALADD cohort was justified by certain clinical similarities with BAs (such as the loss of control over the eating behavior despite the negative consequences), and previous reconciliation in the literature between the two concepts [5–7].

Treatment options and duration is adapted to the evolution of each patient, in a patient-centered approach, and comprises mainly outpatient treatment: psychotropic medications (for psychiatric comorbidities), individual psychotherapies, behavioral and cognitive group therapies, family or couple therapies, support groups (for patients or their family), assessment with a social worker. Inpatient treatment is proposed only when required, for somatic (very low weight, hypokalemia, etc.), psychiatric (high suicidal risk, severe psychiatric comorbidities, etc.) and/or contextual (familial interactions making it difficult to keep the patient from significant harm, etc.) reasons.

The participation to the EVALADD cohort was offered systematically to all incoming patients presenting to seek treatment for either a BA or an ED, and referred to our unit for an outpatient treatment. To be included in the EVALADD cohort, patients must be over 16, have given their written informed consent (and the one of their legal representative if appropriate), be affiliated to Social Welfare, and present with a diagnosis or a subclinical form for one of the 5 disorders studied: problem gambling (PG), sexual addiction (SA), compulsive buying (CB), excessive videogame use (EVU) and eating disorders (ED). Each patient was affected to a unique type of disorder according to his/her main complaint at the beginning of treatment. No patient presented with several main complaints. Subclinical forms represent disorders that do not correspond to all required diagnosis criteria, but with several problems related to the problematic behavior that can justify a need for treatment. A specific assessment was used to perform the diagnosis of each disorder (see Table 1).

**Table 1:** Assessment tools used to perform the diagnosis of each behavioral addiction.

Behavioral addictions	Assessment tools	Threshold used ^a^
Problem gambling (PG)	Interview based on the DSM-IV [14] diagnostic criteria for pathological gambling [15–17]	3/10 [18–20]
Eating disorders (ED)	DSM-IV diagnostic criteria for anorexia nervosa, bulimia nervosa and binge-eating disorder, partly achieved with the Mini International Neuropsychiatric Interview (MINI 5.0) [21]	- *Inclusion of EDNOS*
Excessive videogame use (EVU)	Problem Videogame Playing (PVP) questionnaire [22]	4/9 [23]
Sexual addiction (SA)	Sexual Addiction Screening Test (SAST) [24]	10/25 [24].
Compulsive buying (CB)	McElroy’s criteria [25]	-

^a^ We used lower thresholds than those typically employed to include subclinical forms of behavioral addictions.

Among the 611 patients to whom participation in the study was proposed (41.7% patients with EDs and 58.3% with BAs), about 11% were ineligible to be included in the cohort, and the rate of refusal for eligible patients was very low (around 5%).

### Ethics

The study was conducted in accordance with Good Clinical Practice Guidelines and the Declaration of Helsinki, with approval from the local ethics committee (Groupe Nantais d’Ethique dans le Domaine de la Santé, GNEDS, Nantes). All participants provided written informed consent, including consent from parents or guardians for the participants under age 18. No compensation was given for participation.

### Procedure

As part of the EVALADD procedure, the patients completed a multiaxial psychological assessment through a face-to-face structured interview and self-administered questionnaires. The assessment content was similar for all disorders, in order to allow comparisons. The structured interviews were conducted by trained research staff with experience with BAs and EDs. The assessment was performed at inclusion (just before the beginning of treatment) and then repeated six months, 12 months and each year after inclusion, as long as the participants agree to continue.

Data collection for this study took place between autumn 2009 and spring 2015, with 516 patients included in the cohort. Only patients who completed the inclusion visit (T1), the 6-month follow-up (T2) and the 12-month follow-up (T3) were included in the present analysis. The subsequent follow-up (24 months) gave a loss of patients of almost 60%, and the remaining sample (n = 125) was considered to be too small a sample size to conduct the trajectory analyses. Ultimately, the final database for the present analysis contained 302 patients.

### Measures

Table 2 briefly describes the main variables used in the present study. Measures have been chosen to allow for between-disorders comparisons, i.e. to be used indifferently with all the disorders included in this study.

**Table 2:** Summary of the collected variables exported from the EVALADD cohort (except those used for inclusion).

Scale	Acronym	Utility	Dimensions	Range	I / SAQ^a^	Collected^b^
Number of positive Goodman's criteria (E section) [8]	-	Behavioral addiction severity	-	0 to 9	I	Each visit
Health-related damage	-	Addiction’s impact on patient’s health	-	0 to 5	I	Each visit
Work/school-related damage	-	Addiction’s impact on the patient's professional or educational life	-	0 to 5	I	Each visit
Relationship-related damage	-	Addiction’s impact on the patient's social and family life	-	0 to 10	I	Each visit
Mini International Neuropsychiatric Interview [21]	MINI 5.0	Diagnosis of the main axis-I psychiatric disorders	-	-	I	Baseline and T3
Wender-Utah Rating Scale—Child [26]	WURS-C	Retrospective screening of attention deficit hyperactivity disorder (ADHD) in childhood	-	0 to 100	SAQ	Only at baseline
Adult ADHD Self-Report Scale Symptom Checklist [27]	ASRS-1.1	Screening of attention deficit hyperactivity disorder (ADHD) in adulthood	Inattention Hyperactivity	0 to 36	SAQ	Each visit
Temperament and Character Inventory– 125 [28]	TCI-125	Evaluation of character (epigenetic origin) and temperament (**genetic origin**)	Self-directedness Cooperation Self-transcendence **Novelty seeking** **Harm avoidance** **Reward dependence** **Persistence**	0 to 100	SAQ	Each visit **Only at baseline**
Impulsivity Behavior Scale [29, 30]	UPPS / UPPS-P	Measure of four facets of impulsivity	Negative urgency Lack of premeditation Lack of perseverance Sensation seeking	0 to 16	SAQ	Each visit
Rosenberg Self-Esteem Scale [31]	RSES	Assessment of global self-esteem	-	10 to 40	SAQ	Each visit
Tridimensional Levenson’s locus of control scale [32]	IPAH	Attribution of external or internal causality to events	Internal External-powerful others External-luck	8 to 48	SAQ	Each visit
Defense Style Questionnaire [33]	DSQ-40	Defense mechanisms	Mature Neurotic Immature	1 to 9	SAQ	Only at baseline
Life events [34]	EVE	Measure of the impact of negative life events	-	0 to 400	SAQ	Only at baseline

^a^ I = Interview: the corresponding measure is collected during the face-to-face standardized interview–SAQ = Self-Administered Questionnaires: the corresponding measure is collected through a self-administered questionnaire.

^b^ Each visit denotes that the data are collected at baseline (T1) and at each follow-up (T2 and T3, at 6 and 12 months, respectively).

#### Sociodemographic characteristics

Sociodemographic characteristics were collected: age, sex, marital status, educational level, and employment status.

#### Disorder-related characteristics

Damage. Patients were asked about the extent to which their disorder had caused negative consequences to their life, using a 6-point Likert-type scale. Three different areas were explored: health, work/school and relationships.

Disorder course. The age of initiation of the problematic behavior, the age at which the behavior became problematic, and the age at which the patient first pursued treatment were collected. The patients were asked whether they were able to completely stop the problematic behavior for at least one month, and if so, the maximum abstinence duration was collected.

Addiction severity. Goodman’s addictive disorder criteria were used to assess the addiction’s severity for all of the BAs and the EDs [8]. The use of Goodman’s criteria in EDs have been demonstrated as relevant in previous research [5], except for the restrictive form of anorexia nervosa, for which Goodman’s criteria were considered to be inappropriate (difficulty identifying the beginning and the end of the behavior). In the present study, we used responses to item E as a dimensional score of the addiction’s severity, which can range from 0 to 9.

#### Psychiatric comorbidities

The French version of the MINI 5.0 [21] was used to explore the main axis-I psychiatric disorders at T1 and T3: anxiety disorders, mood disorders (plus the current risk of suicide), addictive disorders and psychotic disorders.

The French version [35] of the Wender-Utah Rating Scale-Child (WURS-C) [26] was used to perform a retrospective diagnosis of Attention Deficit Hyperactivity Disorder (ADHD) in childhood and was supplemented by the French version [36] of the Adult ADHD Self-report Scale (ASRS-v1.1) [27], which screens for ADHD in adulthood.

#### Psychological characteristics

Personality. The Temperament and Character Inventory (TCI-125) [37]was used to rapidly explore four temperament traits (novelty seeking, harm avoidance, reward dependence and persistence) and three character traits (self-directedness, cooperation and self-transcendence). We used the 125-item French version [38].

Impulsivity. The French version [39] of the Impulsivity Behavior Scale (UPPS) [29] was used to measure impulsivity. During the data collection, we moved to the UPPS-P French short version of the scale [30]. To standardize the results, we have reconstructed the four available scores of the new UPPS-P (“negative urgency,” (lack of) “premeditation,” (lack of) “perseverance” and “sensation seeking”) based on the initial UPPS for the first patients.

Self-esteem. The Rosenberg Self-Esteem Scale (RSES) [31] is a 10-item self-report questionnaire that explores the global self-esteem of a person. A validated French version of this scale was used for our study [40].

Locus of control. The French-Canadian version [41] of the Levenson’s questionnaire (IPAH) [32]was used to explore locus of control in three dimensions: internally, powerful others and chance.

Defense mechanisms. The Defense Style Questionnaire (DSQ) [33] explores the predominant defense style for each participant: mature, neurotic or immature. We used the French 40-items version [42].

Negative life events. We used a revised version of the French Life Events questionnaire (EVE) [34], which was previously used in another study on the EVALADD cohort [16]. The revised EVE questionnaire explores 6 areas (family, professional life, social life, marital and emotional life, health, other traumatic events) and allows to compute a total cumulative score of traumatic events by summing the intensity of trauma experienced for each of the 40 items.

### Statistical analysis

**Fig 1:**
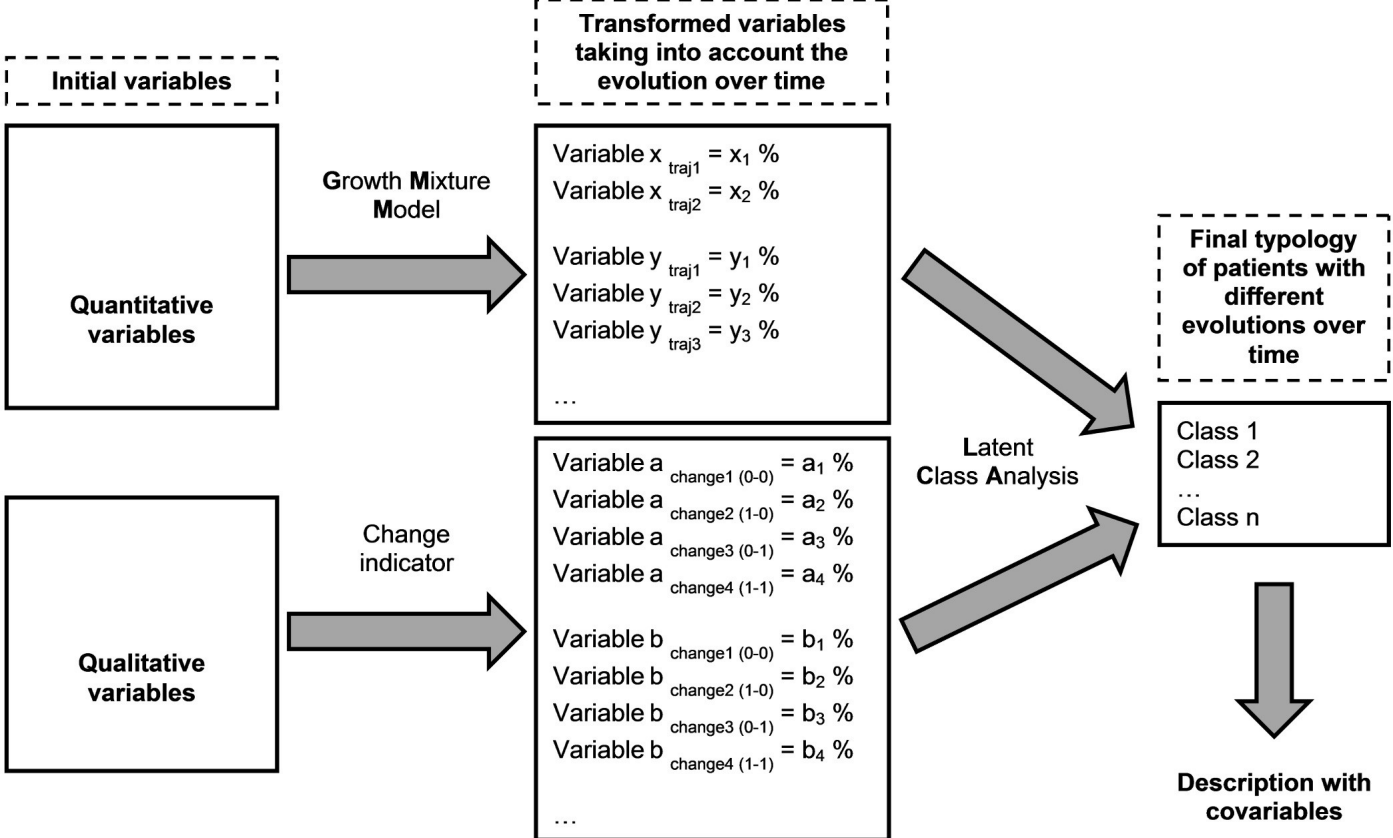
Diagram schematically describing the strategy of the statistical analyses. Caption: Growth Mixture Model (GMM): - x is the first variable used for the GMM. - traj1 and traj2 represent the different trajectories obtained after the GMM analysis for the variable x. - x_1_, x_2_, etc. represent the different assignment probabilities associated with each trajectory for the variable x. Change indicator: - change1 (0–0): the variable is absent at T1 and at T3. - change2 (1–0): the variable is present at T1 and absent at T3. - change3 (0–1): the variable is absent at T1 and present at T3. - change4 (1–1): the variable is present at T1 and at T3.

We used a two-step approach to establish a typology of patients evolving differently over time. The first step was to identify different trajectories of patients for each variable. The second step was to supplement this analysis by a classification grouping together patients with similar profiles of evolution. Finally, we described the obtained classes. The database has been prepared in SAS 9.3, while the different statistical analyzes were carried out with the Mplus 7.3 software [43]. A schematic representation of the strategy used for the statistical analyses is given in Fig 1.

#### Step 1: Identification of trajectories for each variable

We focused only on variables that can evolve over time: (i) scores from the UPPS and the ASRS, the character dimensions of the TCI, and the intensity of damage for continuous variables and (ii) psychiatric comorbidities (MINI) for categorical variables.

For continuous variables, we used Growth Mixture Models (GMMs) through a structural equation modeling framework [44] to identify different patterns of change among a potentially heterogeneous population. For each variable, several models were tested with a varying number of different trajectories (1 to 4), various shape of each trajectory (quadratic, linear, constant), the application of a random effect on the intercept only or on the intercept and slope, and equal or different residuals’ variances. We then followed a backward procedure, going from the most complex model (quadratic shape, different residuals’ variances and random effect on intercept and slope) to the simplest. Step by step, we excluded the trajectories which were not statistically significant according to the Wald test (with type I error fixed at 0.05). We finally obtained a set of several models composed of 1 to 4 trajectories for each variable. To select the most appropriate model for each variable, we used 4 main criteria: the lowest Sample-Size Adjusted Bayesian Information Criteria (SABIC) [45], the sample size of the less represented trajectory of more than 4% of the total sample (n = 13); at least 8 out of 10 models that converged to consider a model to be stable [46] and clinical interpretability and relevance [47, 48]. Outcomes of GMMs are assignment probabilities, which represent the probabilities that a patient will belong to each trajectory composing the model (rather than the assignment of a patient to a unique trajectory). For example, with a model composed of 3 types of trajectories (trajectory A: constant, trajectory B: decreasing and trajectory C: increasing) for a variable X, a patient will be characterized by 3 probabilities of following either trajectory A, B or C. This procedure is particularly interesting for avoiding a loss of information when assigning a patient to a single trajectory while he may transit from one trajectory to another.

For categorical variables (only MINI diagnosis), because it was impossible to apply GMMs, we created a change indicator, making it possible to reflect changes over time. The change indicator was constructed as a categorical variable, which could take 4 different values depending on the 4 possible evolutions between the beginning (T1) and the end (T3) of the follow-up, i.e.: absent at T1 and T3, present only at T1, present only at T3 and present at T1 and T3.

#### Step 2: Classification of patients

We used latent class analysis (LCA) to classify patients within the final typology. We applied the LCA to assignment probabilities obtained from GMMs and change indicators. We tested several models whose number of classes ranged from 2 to 7. To determine the best number of classes, we used the same 4 criteria as for the GMMs. We used assignment probabilities as outcomes for the LCA.

#### Descriptions of classes

We used all of the variables collected, both those used in the trajectory analysis (evolving over time) and the others, to describe the classes. To compare each class to the total sample, we performed analyses of variance (ANOVAs) or non-parametric tests for continuous variables and chi-square or Fisher exact tests for categorical variables. The conventional 5% value was used for significance.

## Results

### Description of the sample

Among the 302 patients included in the analysis, the five disorders were represented in different proportions: ED (43%), PG (35%), SA (14%), EVU (6%) and CB (1%). The proportion of men and women was identical (50%), and the mean age was 34 years. Half of the patients were single (51%), 47% were professionally active, and 36% had an educational level higher than high school graduation. Initiation of the behavior occurred on average at 17 years, and the behavior became problematic approximately 10 years later.

### Growth Mixture Models

Table 3 shows the models selected for each score and graphic illustrations of the obtained trajectories are given in Supporting Information (S1–S4 Figs). All of the models selected followed linear trends and/or constants.

**Table 3:** Models obtained for each selected variable with Growth Mixture Model analysis.

		n	Trajectories forms	Sample size of the smallest trajectory (%)	SABIC	Entropy	Number of converging models	Number of models with highest LL
**UPPS-P**								
	**Negative urgency**	295	2C DV REIS	18.3	3339.496	0.578	10	4
	**Lack of premeditation**	295	2L1C DV REI	11.5	3204.375	0.556	10	6
	**Lack of perseverance**	295	2L1C DV REI	20.3	3188.490	0.497	10	7
	**Sensation seeking**	295	3L1C DV REI	9.9	3436.635	0.550	10	4
**ASRS**								
	**Inattention**	290	2L1C DV REI	8.1	3608.161	0.534	10	4
	**Hyperactivity**	290	2L1C DV REI	7.6	3578.503	0.506	10	5
**TCI**								
	**Self-directedness**	298	4L DV REI	6.6	6284.298	0.766	10	6
	**Cooperation**	298	1L2C DV REI	23.1	5664.110	0.671	10	4
	**Self-transcendence**	298	4L DV REI	12.3	6209.543	0.659	10	7
**Dammage**								
	**Health**	302	2C EV REI	18.9	3157.642	0.753	10	1
	**Work/school**	293	3C EV REI	7.8	2880.42	0.831	10	5
	**Relationship**	302	1L1C DV REI	7.2	3887.075	0.899	10	7

Notes:

LL: log-likelihood

DV: different residual variances / EV: equal residual variances

REIS: random effect on the intercept and slope / REI: random effect on the intercept only

SABIC: Sample-Size Adjusted Bayesian Information Criteria

For each variable, several growth mixture models composed of 1–4 trajectories were tested using a constant (C), a linear (L) or a quadratic (Q) trend for each trajectory.

The numbers before C and L give the number of trajectories of each type. For example, for the “Inattention” score of the ASRS, “2L1C VD EAI” denotes that the selected model comprised 2 linear and 1 constant trajectories, with different residual variances and a random effect on the intercept only.

### Latent class analysis

As shown in Table 4, we selected the 5-class solution, which correctly classified 95.5% of patients.

**Table 4:** Latent class analysis: Properties of the models composed of 2 to 7 classes.

	2 classes	3 classes	4 classes	5 classes	6 classes	7 classes
**SABIC**	4057.1	3177.7	2880.4	2211.0	2619.9	2084.1
**% of patients**						
Class 1	92.1	7.9	7.9	4.3	10.0	14.7
Class 2	8.0	64.5	38.1	30.5	2.6	2.0
Class 3	-	27.6	18.0	17.6	7.6	6.1
Class 4	-	-	36.0	42.0	22.3	16.5
Class 5	-	-	-	5.6	55.5	47.6
Class 6	-	-	-	-	2.0	7.6
Class 7	-	-	-	-	-	5.5
**Entropy**	1.000	0.957	0.942	0.955	0.976	0.971

### Typologies

After comparison of each class to the total sample, we found that one class (n = 92) significantly emerged for the majority of variables, making it impossible to characterize the other classes. Consequently, first, we compared the class with 92 patients to the total sample (= entire sample) and, second, the four other classes to the total sample except the class with 92 patients (= corrected sample). Descriptions of the classes and p-values of the comparisons performed are given in Table 5.

**Table 5:** Description of the 5 classes.

	**Patients with complex psychological functioning** **(n = 92)**			**Patients with impulsive psychological functioning (n = 13)**			**Patients with cooperative psychological functioning (n = 53)**			**Patients with immature psychological functioning (n = 127)**			**Patients with resilient psychological functioning (n = 17)**			**Entire sample** **5 classes**^a^ **(n = 302)**		**Corrected sample** **4 classes**^b^ **(n = 210)**	
**Qualitative variables**	**n**	**%**	**p-value** **5 classes**	**n**	**%**	**p-value** **4 classes**	**n**	**%**	**p-value** **4 classes**	**n**	**%**	**p-value** **4 classes**	**n**	**%**	**p-value** **4 classes**	**n**	**%**	**n**	**%**
**Type of disorder**			0.2448			**0.0354**			0.1353			0.9272			0.1597				
Compulsive buying	1	1.1		0	0		1	2		1	1		1	6		4	1	3	1
Sexual addiction	19	20.7		4	31		7	13		11	9		1	6		42	14	23	11
Problem gambling	22	23.9		7	54		12	22		55	43		11	65		107	35	85	41
Excessive videogame use	7	7.6		1	8		3	6		7	5		0	0		18	6	11	5
Eating disorders	43	46.7		1	8		30	57		53	42		4	24		131	43	88	42
**Sex**			0.9048			**0.0027**			0.1971			0.9535			0.7864				
Women	45	49		1	8		32	60		65	51		8	47		151	50	106	50
Men	46	51		12	92		21	40		62	49		9	53		150	50	104	50
**Marital status**			0.0500			0.0584			0.1053			0.6656			0.7077				
Lives alone	66	72		4	8		38	71		70	56		9	47		187	62	121	58
Lives in couple	24	26		9	92		15	29		56	44		8	53		113	37	89	42
Other	2	2		0	0		0	0		0	0		0	0		2	1	0	0
**Educational level**			0.8923			0.5501			0.4183			0.5026			0.5944				
Under high school graduation	33	36		3	23		15	28		48	38		7	41		106	35	73	35
Over high school graduation	59	64		10	77		38	72		78	62		10	59		196	65	137	65
**Employment situation**			0.1172			0.2388			0.7543			0.7192			0.1026				
Inactive	56	62		4	31		26	50		58	46		11	69		155	52	99	48
Active	35	38		9	69		26	50		67	54		5	31		142	48	107	52
**ADHD**			**<0.0001**			**0.0208**			0.2123			0.7905			0.2761				
No ADHD	36	39		7	54		48	90		102	81		12	71		205	68	169	80
ADHD persistent (adulthood)	39	42		3	23		1	2		6	4		2	12		51	17	12	6
ADHD no persistent (childhood)	17	19		3	23		4	8		19	15		3	18		46	15	29	14
**Total abstinence for at least a month**			**0.0293**			0.3368			0.5075			0.6163			0.8922				
No	64	61		4	36		23	46		67	54		9	53		167	57	103	51
Yes	28	30		7	64		27	54		57	46		8	47		127	43	99	49
**Mood disorders**			**<0.0001**			0.1065			0.1754			0.9165			0.0528				
No	10	11		9	69		19	36		57	45		12	71		107	35	97	46
Yes	82	89		4	31		34	64		70	55		5	29		195	65	113	54
**Anxiety disorders**			**<0.0001**			0.0509			0.5717			0.7896			0.5438				
No	19	21		11	85		28	52		70	55		11	68		139	46	120	57
Yes	73	79		2	15		25	48		57	45		6	35		163	54	90	43
**Alcohol-related disorders**			**0.0020**			0.4770			0.4483			0.9716			0.7547				
No	51	56		9	69		44	84		101	80		13	76		219	73	168	80
Yes	41	44		4	31		9	16		26	20		4	24		83	27	42	20
**Substance-related disorders**			**0.0302**			0.68880			0.7389			0.7444			>0.999				
No	66	72		11	85		45	84		111	87		15	88		248	82	182	87
Yes	26	28		2	15		9	16		16	13		2	12		54	18	28	13
**Antisocial personality disorder**			0.5115			>0.999			0.5865			0.7320			0.3766				
No	88	95		13	100		53	100		123	97		16	94		293	97	205	98
Yes	4	5		0	0		0	0		3	3		1	6		9	3	5	2
**Suicidal risk**			**0.0030**			0.0672			0.6697			0.4055			0.6476				
No	31	34		11	85		34	63		68	54		11	65		155	51	124	59
Yes	61	66		2	15		19	37		58	46		6	35		147	49	86	41
	**Patients with complex psychological functioning (n = 92)**			**Patients with impulsive psychological functioning (n = 13)**			**Patients with cooperative psychological functioning (n = 53)**			**Patients with immature psychological functioning (n = 127)**			**Patients with resilient psychological functioning (n = 17)**			**Entire samplev** **5 classes**^**1**^ **(n = 302)**		**Corrected sample** **4 classes**^**2**^ **(n = 210)**	
**Quantitative variables**	**Mean ± SD**		**p-value** **5 classes**	**Mean ± SD**		**p-value** **4 classes**	**Mean ± SD**		**p-value** **4 classes**	**Mean ± SD**		**p-value** **4 classes**	**Mean ± SD**		**p-value** **4 classes**	**Mean ± SD**		**Mean ± SD**	
**Age**	32.6	±7.7	0.2645	35.4	±2.9	0.8622	34.0	±6.1	0.7034	33.9	±9.5	0.6476	40.7	±4.0	0.1718	34.0	±14.6	34.6	±12.4
**Age of initiation**	14.9	±2.6	**<0.0001**	15.4	±0.7	**0.0464**	18.3	±3.3	0.5983	17.3	±4.7	0.4229	21.2	±2.5	0.2052	16.9	±7.0	17.8	±6.4
**Age of onset of problems**	25.4	±6.6	0.1134	30.8	±2.9	0.4926	26.3	±5.2	0.3051	27.5	±8.1	0.7863	33.0	±3.2	0.1514	27.1	±12.6	27.8	±10.7
**Age of first care**	29.9	±7.7	0.1582	34.4	±3.1	0.6541	30.0	±5.9	0.1745	32.0	±9.8	0.8704	40.5	±4.1	0.0743	31.6	±15.0	32.4	±12.8
**Duration (years)**																			
between initiation and problems	10.5	±6.5	0.7572	14.7	±2.7	0.3110	8.1	±4.2	0.1307	10.2	±7.2	0.8250	11.8	±2.6	0.5217	10.2	±11.2	10.0	±9.2
between problems and care	4.7	±4.1	0.9365	3.6	±0.6	0.2800	3.6	±2.6	0.1788	4.7	±4.3	0.7411	7.5	±2.4	0.2509	4.6	±7.0	4.6	±5.7
between initiation and care	14.9	±7.7	0.8503	18.5	±2.9	0.4219	11.8	±5.1	0.0766	14.7	±8.9	0.8141	19.3	±3.5	0.2083	14.7	±13.7	14.6	±11.3
**Number of Goodman criteria (addiction severity)**	5.4	±2.0	**0.0012**	4.0	±0.7	0.3051	4.3	±1.6	**0.0003**	3.4	±2.3	**0.0019**	4.7	±0.4	0.0905	4.3	±3.7	3.7	±3.0
**WURS-C**	52.8	±9.9	**<0.0001**	37.3	±3.7	0.3530	26.9	±6.4	**0.0091**	33.7	±10.9	0.2642	31.1	±4.5	0.8745	38.3	±19.8	32.0	±14.2
**RSES**	20.7	±2.8	**<0.0001**	26.3	±0.7	0.8304	26.5	±3.1	0.6699	24.6	±3.4	**0.0001**	29.2	±0.5	0.1605	23.9	±6.0	25.5	±4.8
**IPAH**																			
Internal	34.3	±3.0	0.0629	31.3	±0.1	**0.0061**	32.5	±2.1	0.4865	33.0	±3.2	0.9946	32.8	±1.5	0.9688	33.3	±5.2	32.8	±4.2
External—powerful others	30.1	±3.3	**<0.0001**	28.3	±1.3	0.5643	24.2	±3.9	0.5978	27.0	±3.8	**0.0021**	23.7	±1.4	0.7348	27.3	±7.0	26.1	±7.2
External—luck	30.0	±3.8	**<0.0001**	21.5	±1.2	0.6213	22.7	±3.5	0.1663	26.7	±4.1	**0.0008**	23.2	±1.2	0.7373	26.7	±7.4	25.1	±5.9
**DSQ**																			
Mature	5.4	±0.6	0.4008	5.9	±0.3	0.4085	5.5	±0.5	0.5242	5.5	±0.8	0.8776	5.8	±0.3	0.5653	5.5	±1.2	5.6	±1.0
Neurotic	5.3	±0.7	**<0.0001**	4.2	±0.3	0.3239	4.4	±0.5	0.1844	4.8	±0.8	0.1571	4.5	±0.3	0.6841	4.9	±1.3	4.7	±1.0
Immature	5.2	±0.5	**<0.0001**	4.2	±0.3	0.8045	3.6	±0.4	**<0.0001**	4.3	±0.7	**0.0288**	4.0	±0.2	0.6951	4.4	±1.1	4.1	±0.9
**Life events score (cumulative traumatic intensity)**	56.0	±21.6	**0.0208**	55.0	±5.0	0.5406	39.5	±13.7	0.5577	44.2	±18.9	0.4390	15.0	±1.3	**0.0004**	46.3	±33.4	41.6	±24.5
**Number of life events**	10.3	±3.2	0.9001	8.6	±0.6	0.6577	8.6	±2.2	0.3988	9.6	±3.5	0.3349	8.7	±1.2	0.5035	9.5	±5.4	9.2	±4.4
**TCI**																			
Novelty seeking	59.3	±11.3	**<0.0001**	60.8	±4.4	**0.0399**	38.8	±5.6	**0.0001**	46.0	±12.5	0.8824	53.5	±3.5	**0.0416**	49.9	±20.2	45.7	±15.5
Harm avoidance	67.1	±13.6	**0.0116**	46.7	±4.2	0.0695	58.9	±10.9	0.8834	61.3	±15.8	0.3634	54.4	±5.3	0.3840	61.7	±24.9	59.3	±20.6
Reward dependence	57.8	±9.1	0.0779	55.0	±3.2	0.2057	63.5	±8.0	0.4147	60.6	±12.6	0.6743	66.3	±5.0	0.3625	60.3	±18.7	61.5	±16.2
Persistence	47.5	±17.0	**0.0008**	46.7	±6.0	0.1447	59.7	±13.0	0.7980	63.0	±19.7	0.3753	57.6	±6.6	0.6528	56.6	±31.1	60.7	±25.4
Self-directedness	37.6	±9.1	**<0.0001**	66.3	±4.2	0.5521	61.4	±8.7	0.6956	55.0	±11.3	**<0.0001**	64.2	±4.7	0.7188	51.7	±20.7	58.1	±15.9
Cooperation	68.8	±9.4	**<0.0001**	72.3	±2.9	0.1435	83.9	±4.4	**0.0009**	75.4	±10.1	**0.0087**	79.1	±4.0	0.9807	74.9	±16.2	77.7	±12.5
Self-transcendence	39.1	±12.3	**0.0005**	27.2	±4.5	0.9470	23.8	±8.6	0.2544	31.4	±13.5	**0.0128**	29.4	±5.4	0.6445	32.2	±22.0	29.1	±17.7
**Maximum abstinence duration (months)**	3.5	±1.7	0.2534	1.9	±0.2	**<0.0001**	4.8	±3.4	0.8450	4.7	±5.0	0.7950	3.0	±0.5	**0.0210**	4.2	±6.4	4.4	±6.1
**UPPS-P**																			
Negative urgency	12.0	±1.4	**<0.0001**	11.4	±0.5	**0.0122**	6.7	±0.7	**<0.0001**	10.6	1.7	**<0.0001**	11.4	±0.7	**0.0123**	10.4	±3.1	9.7	±2.5
Lack of premeditation	9.6	±1.5	**<0.0001**	9.4	±0.3	**0.0007**	7.0	±0.8	**0.0059**	7.9	1.4	0.2817	8.3	±0.7	0.3462	8.3	±2.5	7.8	±1.9
Lack of perseverance	9.5	±1.4	**<0.0001**	7.7	±0.4	0.4744	7.2	±1.0	0.8826	7.2	1.3	0.9309	7.2	±0.5	0.9802	7.9	±2.5	7.2	±1.7
Sensation seeking	11.4	±1.6	**<0.0001**	11.2	±0.5	**0.0117**	8.4	±1.3	0.0770	9.8	1.8	**0.0050**	10.4	±0.5	**0.0250**	10.1	±3.0	9.6	±2.4
**ASRS**																			
Inattention	23.7	±3.1	**<0.0001**	15.6	±1.0	0.4113	14.5	±2.2	0.9986	14.7	2.8	0.6740	15.5	±1.8	0.5746	17.6	±6.6	14.7	±4.1
Hyperactivity	19.2	±3.1	**<0.0001**	14.8	±0.9	0.2360	12.4	±2.4	0.3868	14.2	3.7	0.0536	12.5	±1.2	0.6408	15.4	±6.2	13.6	±4.7
**Damages**																			
Health (/5)	3.8	±0.6	**<0.0001**	1.6	±0.3	0.2141	2.9	±0.7	**0.0002**	3.1	1.0	**<0.0001**	2.1	±0.4	0.9491	3.1	±1.5	2.9	±1.3
Work/school (/5)	2.1	±1.1	**0.0006**	0.8	±0.3	0.9986	1.7	±0.8	**0.0006**	1.1	1.0	**0.0302**	0.4	±0.2	0.2165	1.4	±1.8	1.2	±1.4
Relationship (/10)	7.3	±1.2	**<0.0001**	1.8	±0.1	**<0.0001**	6.1	±1.0	**<0.0001**	6.2	1.3	**<0.0001**	0.4	±0.1	**<0.0001**	6.0	±2.7	5.4	±2.3

^a^ Total-5 classes corresponds to the entire sample.

^b^ Total-4 classes corresponds to the entire sample except the “complex patients” class, i.e. the “corrected sample”.

#### Patients with complex psychological functioning

This class contained 92 patients (30%), and significantly emerged from the others, with the majority of the variables being significantly different from the entire sample. The sociodemographic characteristics and distribution of disorders did not differ from the entire sample.

This class displayed the highest rate of inability stopping the problematic behavior for at least one month (61%), largely over the other classes. The age of problematic behavior onset was lower (15 years), with no other difference in the problematic behavior course.

This class was characterized by a more severe symptomatology, with a higher addiction severity and a higher level of disorder-related damage in all domains. This high-severity profile was associated with multiple comorbidities (mood disorders, anxiety disorders, substance use disorders and ADHD) and numerous psychopathological personality traits (low self-esteem, high neurotic and immature defense mechanisms, a high external locus of control, high impulsivity, high novelty seeking and harm avoidance temperaments, a low persistence temperament, low self-directedness and cooperation character traits, and a high transcendence character trait). They experienced a very high rate of suicidal risk (two-thirds of the class) and displayed a higher negative life events score.

All of these arguments led us to define these patients as patients with complex psychological functioning to illustrate their high level of severity in all psychopathological domains explored.

After the exclusion of patients with complex psychological functioning, the corrected sample used for the comparative analyses of the other four classes was reduced to 210 patients.

#### Patients with impulsive psychological functioning

This class was the smallest, with only 13 patients (4%). These patients presented mostly with PG and SA, and ED were largely underrepresented compared to the other classes.

Men were overrepresented, and the average age of initiation was low. Their internal locus of control score was lower, but the difference did not reach clinical relevance (one-point difference).

We identified an impulsive profile because of three arguments: a higher novelty seeking score, three higher impulsivity scores (lack of premeditation, negative urgency and sensation seeking) and more frequent histories of ADHD. Despite having the highest experimentation of abstinence, patients with impulsive psychological functioning appeared to be less able to durably stop their problematic behavior, with a significantly lower maximum duration of abstinence (half of the entire sample). An unexpected result was that the problematic behavior had less impact on relationships.

#### Patients with cooperative psychological functioning

This class was composed of 53 patients (18%). The sociodemographic characteristics, distribution of disorders, problematic behavior course and comorbid psychiatric disorders within this class did not differ from the corrected sample. Patients with cooperative psychological functioning were found to have a higher addiction severity than the corrected sample, although this difference was very small (0.6 points out of 9). They displayed a lower childhood ADHD score, a lower immature defense style score and a lower novelty seeking score. They were also characterized by a low level of impulsivity, particularly on the negative urgency and lack of premeditation dimensions. The attribute that best characterized this class was a significantly higher cooperation score. Finally, they declared a higher impact of the problematic behavior, although to a lower extent than for complex patients.

#### Patients with immature psychological functioning

This class was the most represented, with 127 patients (42%). There were no differences in the sociodemographic characteristics, distribution of disorders, problematic behavior course and comorbid psychiatric disorders. This class had the lowest addiction severity. These patients displayed a lower self-esteem score, a lower cooperation score and a higher transcendence score. They also presented a high impulsive profile, particularly on the negative urgency dimension. The levels of reported damage were lower for the work/school domain and higher for health and relationships domains, but the difference was clinically relevant only for relationships. We identified this class as being patients with immature psychological functioning because of a higher external locus of control, a lower self-directedness score and a higher immature defense style score.

#### Patients with resilient psychological functioning

This class was the second smallest class, with 17 patients (6%). There were no differences for the sociodemographic characteristics, distribution of disorders, problematic behavior course and comorbid psychiatric disorders. Patients with resilient psychological functioning were high novelty seekers and displayed high levels of impulsivity on the negative urgency and sensation seeking dimensions. They experienced a lower maximum duration of abstinence, although higher than for impulsive patients. We identified the patients from this class as having a resilient psychological functioning because it seemed that they were more efficiently able to cope with problems. One illustration of this point is the low reported level of negative consequences, despite an equivalent or even higher (although not significant) level of addiction severity. Another illustration is the fact that the negative life events score was three times lower than for the other classes, despite an equivalent number of experienced life events and no difference in age.

**Fig 2:**
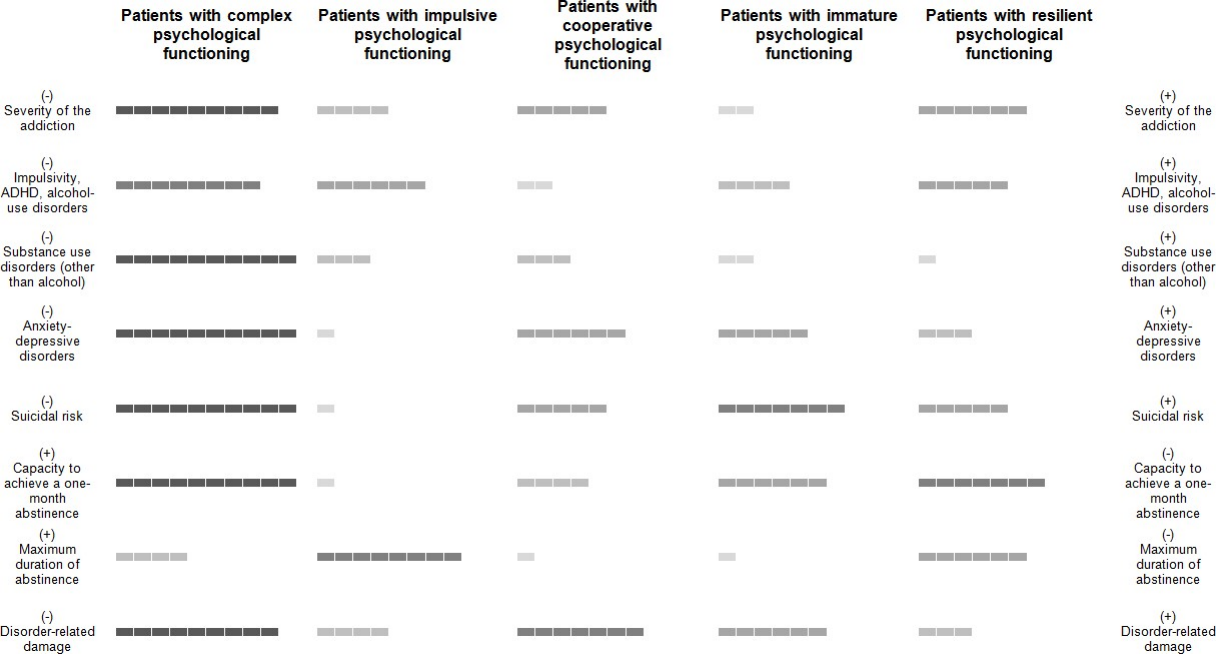
Schematic representation of the five types of patients with behavioral addictions or eating disorders. (+) High level of the characteristic (relative to the other classes). (-) Low level of the characteristic (relative to the other classes). The (+) and (-) have been positioned to indicate a high level of psychopathology on the right column and a low level on the left column. For example, patients with impulsive psychological functioning have a high capacity to achieve a one-month period of abstinence, which was associated with a low level of psychopathology, but a low capacity to maintain abstinence (low duration), which was associated with a high level of psychopathology. Patients with complex psychological functioning presented with the highest severity, the highest disorder-related damage, the highest level of psychiatric and addictive comorbidity, the highest suicidal risk, the highest level of impulsivity, and the lowest capacity to achieve a one-month period of abstinence, giving a multiple-psychopathological profile.

Fig 2 is an attempt to summarize the main characteristics of each class.

## Discussion

Trajectory analyses have rarely been used in the context of BAs, except for some studies on PG [10, 12], and have been little used for EDs [11, 13]. To the best of our knowledge, our study is the first to use growth mixture modeling followed by latent class analysis, making it possible to identify five classes of patients with BAs or EDs characterized by different trajectories during a one-year period of specialized care. This kind of approach has lead both to discuss the similarities of the clinical trajectories between the BAs with each other and with EDs, and to clinical implications for care management.

### Similarities of the clinical trajectories between the BAs with each other and with EDs

As stated in the introduction, we expected to obtain one of the following typologies: (i) a common care trajectory for all studied disorders (both BAs and EDs), (ii) a distinct trajectory for each type of disorder, or (iii) different trajectories independent of the type of disorder. The last hypothesis was confirmed, with a final typology not linked to the type of disorder, but rather, based on the profiles of patients. Indeed, we did not identify any significant grouping of disorders within any class of the typology. The only class that demonstrated a differential repartition of disorders was the “impulsive patients” class, which was also the least represented class. It should be noted that, despite the fact that EVU and EDs are not consensually included in the framework of addictions, these disorders did not group separately from the other BAs. A previous study demonstrated that a subgroup of individuals with EDs displayed an “addictive-like” ED and share some addictive personality traits with substance-related disorders [5]. Our study supports this statement on a longitudinal perspective, by showing common trajectories between EDs and BAs, even for the restrictive type of anorexia nervosa which was found to have lower rates of “addictive-like” ED [5]. Moreover, the 11th revision of the International Classification of Diseases (ICD-11) has recently proposed to include “gaming addiction” under the “disorders due to addictive behaviors” category, thus supporting EVU belongs to the spectrum of BAs [49], even if debate is still open on the level of evidence [50]. As for EDs, our result of common clinical trajectories between EVU and other BAs seems to support the ICD-11 view, although further studies are needed to compare those trajectories with other addictions such as substance-related disorders. In conclusion, the absence of a grouping of the different disorders in the typology allows us to envisage a transversality of the evolutionary profiles between the different forms of BAs and EDs. Therefore, this suggests the relevance of a common addictive concept applicable both to all BAs, including those for which the inclusion under the addictive disorder label is not consensual (such as problematic videogame use or sexual addiction), and also to EDs.

The second conclusion of this work is that there was no clear overlap of the obtained typology with previous theoretical models of BAs or EDs, based on psychological characteristics. For EDs, the theoretical model of eating disorder development is referred as the dual pathway model [51, 52]. However, such model mainly focused on ED symptomatology (dietary restraint and bulimic symptoms), which does not allow for comparisons with other disorders. Furthermore, other works aiming at identifying longitudinal ED trajectories or pathways [11, 13] also explored ED symptoms (variation of weight, change in dietary or eating behaviors, body shape concerns, etc.), rather than psychological characteristics or psychiatric comorbidities. To the best of our knowledge, such a model based on psychological vulnerabilities not directly related to the symptomatology of the disorder only exists in the framework of PG, with the well-known pathway model [53]. This model presumes that there are three distinct types of problem gamblers, with implications for clinical management. Behaviorally conditioned problem gamblers are characterized by the absence of any premorbid psychopathology. Emotionally vulnerable problem gamblers are characterized by premorbid anxiety and/or depression, poor coping skills and negative life events. Antisocial/impulsivist problem gamblers are highly disturbed individuals, with high levels of impulsivity and maladaptive behaviors. If some features of this model can partially overlap with the present typology, this type of theoretical typology does not seem to be applicable to all disorders included in this study. As a consequence, we think that the proposed typology in five classes may provide additional knowledge on the typical pathways of patients with BAs and EDs.

### Clinical implications for the management of BAs and EDs

#### General implications

As stated in the introduction, given that we obtained different trajectories not linked to the type of disorder but, rather, based on patient profiles (profiles of personality, psychiatric comorbidities, etc.), it is possible to envisage the use of common therapeutic tracks independently of the disorder, targeted on individual vulnerabilities (personalized medicine), rather than care only centered on the problematic behavior. This may allow achieving a subject-centered rather than an object-centered clinical approach. Care management of BAs and EDs may benefit from a dual-approach based on the use of therapies that has demonstrated relatively good efficiency for the various addictive and eating disorders (motivational interviewing and cognitive-behavioral therapies (CBT), for example), supplemented by specific approaches focusing on the individual vulnerabilities of each profile from the typology. This may allow patients to partly distance themselves from their problematic behavior and to work on the mechanisms underlying their addictive vulnerability. Consequently, addiction care providers should feel legitimate in managing the various addictive and eating disorders, and addictology departments receiving patients suffering from all types of addictions or eating disorders should be promoted.

#### Clinical implications of the five-class typology

Patients with complex psychological functioning were deeply affected in various domains. Such patients may demonstrate poorer responses to care and less compliance. The complexity of patients in health care has received increasingly more attention from care providers [54, 55]. Achieving congruence between the patient and the care provider is of crucial importance for effective, patient-centered care [54]. The difficulty lies in the management of the multiplicity of associated disorders, together with psychopathological personality traits and negative life events. A possible approach would be to first direct the therapeutic work more to the psychiatric comorbidities, which seem to be very pregnant in this class, rather than to the problematic behavior itself. Indeed, psychiatric comorbidities have been found to increase the risk of relapse and to worsen the prognosis in other substance-related addictive disorders [56], so that appropriate treatment of such comorbidities can significantly improve long-term prognosis and functioning in multiple domains. As a consequence, treating psychiatric comorbidities in patients with complex psychological functioning should be considered a key goal andmay secondarily improve the other psychopathological characteristics (self-esteem, abstinence capacity) and their intrinsic motivation to change, and help the patient be more receptive to addictive care in a second time, in a virtuous cycle of recovery. Because they display a very high rate of suicidal risk and a high level of disorder-related damage, harm reduction should also be provided to limit the negative consequences of the behavior. Finally, mental health providers should keep in mind that access to addiction treatment for these patients with multiple comorbidities may be done through the co-occurring disorders. A systematic screening of addictions (substance-related or not) and EDs should be promoted for all patients presenting for a mental health problem.

Patients with impulsive psychological functioning unexpectedly reported lower damage from the disorder. This finding may be due to a low addiction severity or the fact that they have less awareness of the negative impacts on their lives (lower insight). This class was the smallest one, which may indirectly indicate that they have more difficulty accessing specialized care, especially due to a lower awareness of disorder-related damage. Such a profile of patients does not seek for care easily but may benefit from impulsivity-centered care, focused on the reduction of stress reactivity or the enhancement of coping strategies with psychotherapies and/or the regulation of their biologically-based impulsivity with psychotropic medications [53]. This would be the first step toward favoring care compliance and the durability of abstinence.

In contrast, patients with cooperative psychological functioning reported a slightly higher severity of the addiction, which could reflect a higher awareness of the negative impacts on their lives (higher insight). This could represent a therapeutic lever for these patients because their high cooperativeness may lead them to attempt to solve their problems. Solution-oriented therapies focused on the experienced damage may thus represent an efficient care strategy for these patients.

Patients with immature psychological functioning displayed global weak maturity characterized by a lower ability to appropriately adapt behavior to the situation and the attribution of the causality of events to external causes. Care should focus on the correction of external attribution with the reinforcement of self-esteem and personal skills and on the enhancement of coping strategies.

Finally, patients with resilient psychological functioning were characterized by presumed good coping skills, with a low level of reported damage and negative impact from traumatic events. Such patients may benefit from shorter interventions such as support groups (such as Gamblers Anonymous) or CBT, supplemented by relapse prevention programs.

### Limitations

Some limitations of this study should be noted. First, because the patients with complex psychological functioning class was marked by an extreme level of pathology, it has erased the characteristics of the four other classes. We have attempted to overcome this problem by conducting two separate analyses to describe the classes, with and without these patients. However, it is debatable whether this decision has brought biased results. Second, certain classes (patients with impulsive and resilient psychological functioning) or clinical groups (compulsive buyers) sample sizes were low, which limits the interpretability. Third, we included only patients seeking care and with three time points of evaluation, which could have resulted in a selection bias. Indeed, it is well-known that help-seeking is very low in patients suffering from addictions, both substance-related or not [57, 58]. Moreover, it is assumed that some types of patients, such as impulsive patients, are not very compliant with care [53]. By definition, these patients would therefore be unlikely to be included in our study. Fourth, we have mainly focused on individual vulnerabilities rather than on environmental vulnerabilities, which may have had an impact on the typology obtained and should be taken into account for care management. Moreover, some individual vulnerabilities or frequent comorbid conditions, such as personality disorders, have not been assessed. These choices were made because of the long duration of the EVALADD interviews and the time needed to complete questionnaires at home. The clinical characteristics investigated have thus been reduced to a minimum acceptable both in terms of clinical relevance and duration of assessment, to ensure acceptability of the procedure from patients and especially to maximize the follow-up retention rate. Finally, we made the choice to include all EDs, EVU and subclinical disorders within the framework of this work. As stated in the introduction, these disorders are not consensually linked to the concept of addiction. However, the fact that the typology did not identify these disorders separately could indicate that separating them is no more legitimate than bringing them together.

## Conclusions

To the best of our knowledge, this study is the first attempt to determine the typology of patients with behavioral addictions or eating disorders in a large range of disorders and based on a one-year period of evolution. The typology obtained brings interesting findings to propose patient-centered care strategies adapted to these profiles. Because the typology was independent from the type of disorder, it supports the general concept of addiction for all BAs, including several disorders such as gambling disorder, sexual addiction, and compulsive buying as well as excessive videogame use. It also supports the relevance of exploring the addictive nature of eating disorders, and the overlap between the two categories of disorders. The relevance of this model should be further examined in future studies.

## Supporting information

S1 Fig Graphic representations of the latent trajectories obtained for the 4 UPPS-P dimensions after the Growth Mixture Models analysis. (DOCX)

S2 Fig Graphic representations of the latent trajectories obtained for the 2 ASRS dimensions after the Growth Mixture Models analysis. (DOCX)

S3 Fig Graphic representations of the latent trajectories obtained for the 3 character dimensions of the TCI after the Growth Mixture Models analysis. (DOCX)

S4 Fig Graphic representations of the latent trajectories obtained for the 3 damage scores after the Growth Mixture Models analysis. (DOCX)
