# Supplementary material for: Typology of patients with behavioral addictions or eating disorders during a one-year period of care: Exploring similarities of trajectory using growth mixture modeling coupled with latent class analysis
Source: PLoS One. 2018 Nov 14;13(11):e0207398. doi: 10.1371/journal.pone.0207398 (PMC6235397; doi:10.1371/journal.pone.0207398)
Supplement: S1 Fig — (DOCX) [file pone.0207398.s001.docx]

## S1 Fig. Graphic representations of the latent trajectories obtained for the 4 UPPS-P dimensions after the Growth Mixture Models analysis

|  |  |
| --- | --- |
|  |  |

Note: %=proportions of patients in each trajectory
